# Changes in psychological distress among Polish medical university teachers during the COVID-19 pandemic

**DOI:** 10.1371/journal.pone.0278311

**Published:** 2022-12-01

**Authors:** Bartosz Wojtera, Nisha Singh, Sofia Iankovitch, Lilly Post, Alhassan Ali Ahmed, Mohamed Abouzid

**Affiliations:** 1 Franciszek Raszeja City Hospital in Poznań, Poznań, Poland; 2 Poznan University of Medical Sciences, Poznan, Poland; 3 Department of Bioinformatics and Computational Biology, Poznan University of Medical Sciences, Poznan, Poland; 4 Doctoral School, Poznan University of Medical Sciences, Poznan, Poland; 5 Department of Physical Pharmacy and Pharmacokinetics, Poznan University of Medical Sciences, Poznan, Poland; Julius-Maximilians-Universität Würzburg, GERMANY

## Abstract

Our study aims to update knowledge about psychological distress and its changes in the Polish group of academic medical teachers after two years of a global pandemic. During the coronavirus disease, teachers were challenged to rapidly transition into remote teaching and adapt new assessment and evaluation systems for students, which might have been a completely novel situation that was not addressed before, especially in medical universities in Poland. We conducted a cross-sectional study at Poznan University of Medical Sciences from March to April 2022. The questionnaire included self-reported information on anxiety, stress, and depression. We found that post-pandemic levels of anxiety, stress, and depression have significantly (p<0.001) improved compared to initial levels at the beginning of coronavirus disease. In multivariate models, females had higher odds of improving levels of anxiety (OR  =  0.46; 95% CI  =  -1.58–(-0.03); p  =  0.04), stress (OR  =  0.36; 95% CI  =  -1.83–(-0.22); p  =  0.01), and depression (OR  =  0.0.37; 95% CI  =  -1.58–(-0.12); p  =  0.03). Anxiety, stress, or depression were not significantly associated with years of experience, the number of taught subjects, and weekly teaching hours, but only with the academic work during COVID-19 (Spearman r_anxiety_ = 0.37, r_stress_ = 0.32, r_depression_ = 0.37, p<0.001). For the virtual learning concerns, 79% of teachers reported that students might engage less; and it was correlated with higher weekly teaching hours (r = 0.19, p<0.05). Even though only 29.8% reported cheating as a concern, it was correlated with a higher number of taught subjects (r = 0.2, p<0.05). Levels of anxiety, stress, and depression have improved as time passed, not affecting teachers’ academic performance. Concerns about virtual learning have been raised, suggesting it may be conjoined with classroom learning but not as an alternative. Universities should highlight the importance of seeking psychological support and provide essential programs to employees. Teachers’ coping skills with psychological distress should be further studied.

## 1. Introduction

Coronavirus disease spread rapidly during the first few months of 2020, making the World Health Organization (WHO) announce the coronavirus 2019 (COVID-19) pandemic. According to WHO statistics, the number of infected people until 2022 reached almost 300 million, whereas over 5 million people died; however, the number of undiagnosed cases may be significantly higher [1]. The pandemic reality, national restrictions, and social distancing regulations changed almost all everyday life aspects worldwide, including higher education institutions [2, 3]. As a result, teachers and professors moved to virtual learning quickly, which raised levels of stress and eventually accumulated symptoms of fatigue, anxiety, and psychological distress [4].

Before the COVID-19 pandemic, university teachers were afflicted by significant stress [5]. In 2020, besides general pandemic threats, academic teachers were challenged to rapidly transition into remote teaching and adapt new systems of assessment and evaluation of students, which might be a completely novel situation. Scientific research and international collaboration also were significantly impeded [6, 7].

Most studies concerning psychological distress in academic teachers were published in 2020, during the first few months of the pandemic [8]. According to a systematic review and meta-analysis by Ozamiz-Etxebarria et al. [3], anxiety, depression, and distress were present in all teachers, 17%, 19%, and 30%, respectively. Academic teachers experienced more stress than school teachers, but they experienced anxiety less frequently. The anxiety was significantly higher on the Asian continent. Meta-analysis showed no significant differences in psychological symptoms related to the pandemic distress depending on sex or age, although some studies had reported them. The methodology–using different scales or different ways of data collection had a significant impact on results [3].

The passage of time and frequent restriction changes may significantly impact people’s attitudes, affecting their fears, beliefs, coping strategies, everyday routines, and other issues. Also, studies concerning psychological distress among academic medical teachers are underrepresented. Therefore, our study aims to update knowledge about psychological distress and its changes in a group of medical academic teachers in Poland after two years of a global pandemic, which according to our knowledge, no similar study has been performed.

This study will therefore address the following research questions:

- What are the differences between anxiety, stress, and depression levels from November 2019 (the beginning of COVID-19) till April 2022?
- What is the impact of demographic parameters (gender, years of teaching experiences, number of taught subjects, weekly teaching hours, experience with virtual learning before COVID-19, having psychological distress before COVID-19, and major life-changing events due to COVID-19) on the levels of anxiety, stress, and depression?
- What are the correlations between major online learning concerns and demographic parameters (gender, years of teaching experiences, number of taught subjects, weekly teaching hours, experience with virtual learning before COVID-19, having psychological distress before COVID-19, and major life-changing events due to COVID-19)?
- What are the predictors for improved anxiety, stress, and depression levels?

## 2. Methods

### 2.1. Study design

We performed a cross-sectional study using an anonymous, self-administered and structured online survey tool through the "Microsoft Forms" platform.

### 2.2. Study population

The inclusion criteria were applied to all individuals who agreed to participate in the study: age ≥ 18 years, hold a Ph.D. or M.D, and be a research and teaching staff at Poznan University of Medical Sciences (PUMS). There were no restrictions on gender, age, nationality, or socioeconomic level. The exclusion criteria were all participants less than 18 years, who refused to participate in the study or inaccurately filled the survey.

### 2.3. Sampling

Participation in the survey was promoted in March-April 2022 over a mailing list reaching all members of Poznan University of Medical Sciences (PUMS), followed by repeated reminders directed to the group of professors. According to the PUMS website, the research and teaching staff include 1200 individuals. All 144 respondents who fulfilled the inclusion criteria were used in the analysis.

### 2.4. Study tool

The survey is composed of three parts: (1) the demographic characteristics (age, gender, teaching years of experience, number of subjects taught, number of weekly teaching hours, psychological distress prior to the pandemic (yes/no), prior experience with remote techniques (yes/no), major life changes due to COVID-19); (2) subjective level of anxiety, stress, and depression reported as a 1–5 scale of normal, mild, moderate, severe and extremely severe (when COVID-19 was first discovered and at time of the study); (3) predictors of psychological distress (academic work during COVID-19, the need for psychological support, sleep disturbances, COVID-19 restriction cancellations, repeated canceling and reimplementation, passing time, implementation of well-organized remote teaching, adapting to virtual learning, vaccination, infection status, engaging in unhealthy behaviors and main concerns of remote learning). The survey is available in supplementary files.

### 2.5. Statistical analysis

We performed the statistical analysis using Statistica version 13 coupled with Plus Kit version 3. Shapiro–Wilk test was used to measure the normality of continuous data. Categorical data were reported as frequency/percentage and continuous data as mean/standard deviation (SD) (for normal distribution and scales of anxiety, stress, and depression) or median/interquartile range (IQR) (for non-normal distribution). Intra-differences between variables (e.g. initial anxiety levels vs. current anxiety levels for females) were tested using a non-parametric Wilcoxon signed-rank test with Bonferroni correction. Chi-square with Bonferroni correction tested the inter-differences between the groups (i.e., initial anxiety levels between males vs. females). Moreover, Mann–Whitney U tested anxiety, stress and depression differences between two groups (gender, years of experience, previous experience with online learning and having psychological distress before COVID-19). Moreover, Spearman’s rank correlation coefficient (r) and phi coefficient (Φ) were used to investigate the correlation between several demographic factors and online learning concerns; and between psychological distress predictors and stress, depression and anxiety. Finally, we built three multifactorial backward stepwise logistic regression models for anxiety, stress, and depression; results are presented as odds ratios (ORs) and 95% confidence intervals (95% CI). A p-value less than 0.05 is statistically significant in all tests except in Tables 2 and 3 due to applying Bonferroni correction, where p-values less than 0.00104 and 0.00119, respectively, are considered statistically significant.

## 3. Results

### 3.1 Demographic characteristics of the participants

A total of 144 responses have been collected. The average age of the participants was 44.85 ± 10.78, and there were 64.6% females and 35.4% males. The teachers had an average of 17.53 ± 11.33 years of experience, and the majority were teaching less than five subjects and had from five up to 15 teaching hours per week. Only 29.2% reported facing psychological distress before COVID-19, and only 16.7% had previous experience with online learning. Almost 80% declined to have had any major life-changing events due to COVID-19. Demographic data are presented in (Table 1).

**Table 1:** Demographic data of the participants, frequencies reported as N (%).

**Age**	44.85 ± 10.78; [44 (37–53)]*
**Gender**	
Female	93 (64.6)
Male	51 (35.4)
**Teaching years’ experience**	17.53 ± 11.33; [(16 (7–25)]*
< 20 years	81 (56.3)
≥ 20 years	63 (43.8)
**Number of teaching subjects**	3.81 ± 3.25; [(3 (2–5)]*
< 5 subjects	95 (66)
≥ 5 subjects	49 (34)
**Weekly teaching hours**	
5 to < 10 hours a week	53 (36.8)
10 to < 15 hours a week	53 (36.8)
15 to < 20 hours a week	19 (13.2)
≥ 20 hours a week	19 (13.2)
**Psychological distress before COVID-19**	
No	102 (70.8)
Yes	42 (29.2)
**Previous experience with online learning**	
No	120 (83.3)
Yes	24 (16.7)
**Major life-changing due COVID-19**	
No	115 (79.9)
Yes	29 (20.1)

* reported as mean ± SD; [median (IQR)]*

### 3.2 Initial and current levels of anxiety, stress, and depression

**Fig 1:**
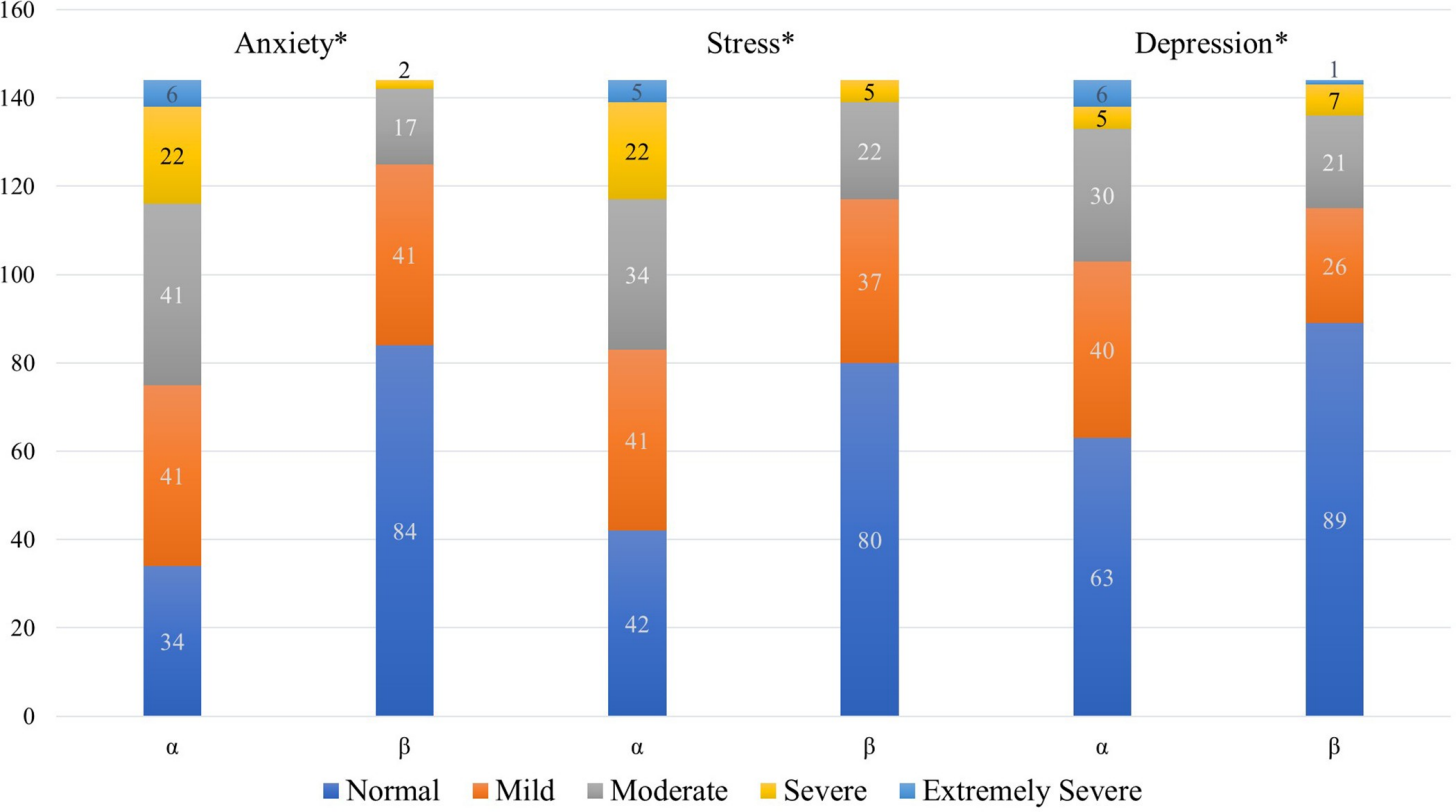
Subjective ratings of level of anxiety, stress, and depression when COVID-19 was first discovered (retrospective rating) and after COVID-19-Pandemic restrictions were lifted.

Overall, anxiety, stress, and depression levels after two years of the pandemic were significantly lower than the initial levels (when COVID-19 started) (Fig 1).

Females showed significant improvement in anxiety; ratings changed from 2.66 = between "mild" and "moderate" at pandemic onset to 1.55 after pandemic restrictions were lifted = between "normal" and "mild". Stress levels were reduced by 0.9, from between "mild" and "moderate" to between "normal" and "mild". Depression levels changed from 2.09 = between "mild" and "moderate to 1.63 = between "normal" and "mild".

Males reported numerically higher anxiety ("mild" < 2.16 < "moderate" vs. "normal" < 1.59 < "mild"), stress levels ("mild" < 2.04 < "moderate" vs. "normal" < 1.73 < "mild"), and depression ("normal" < 1.75 < "mild" vs. "normal" < 1.67 < "mild") at the pandemic onset than when the study occurred, however, the differences were insignificant (Table 2).

**Table 2:** Intra-differences analysis between initial and current levels of anxiety, stress, and depression using paired Wilcoxon signed-rank test with Bonferroni-correction.

		Initial level		Current level			
**Demographic parameter**		Mean	Median	Mean	Median	**z**	**p**
		(SD)	(Q1-Q3)	(SD)	(Q1-Q3)		
**Anxiety**							
**Gender **	Female	2.66 (1.14)	3 (2–3)	1.55 (0.76)	1 (1–2)	6.69	<0.001*
	Male	2.16 (1.07)	2 (1–3)	1.59 (0.75)	1 (1–2)	3.25	0.001
**Teaching experience (yrs.) **	< 20 years	2.6 (1.14)	3 (2–3)	1.56 (0.81)	1 (1–2)	5.87	<0.001*
	≥ 20 years	2.32 (1.12)	2 (1–3)	1.57 (0.69)	1 (1–2)	4.54	<0.001*
**Subject No. **	< 5 subjects	2.54 (1.16)	2 (2–3)	1.6 (0.75)	1 (1–2)	6.18	<0.001*
	≥ 5 subjects	2.37 (1.09)	2 (1–3)	1.49 (0.77)	1 (1–2)	4.13	<0.001*
**Weekly teaching hours (hrs.) **	5 to < 10	2.57 (1.03)	3 (2–3)	1.62 (0.81)	1 (1–2)	4.66	<0.001*
	10 to < 15	2.42 (1.18)	2 (1–3)	1.53 (0.72)	1 (1–2)	4.41	<0.001*
	15 to < 20	2.42 (1.17)	3 (1–3)	1.42 (0.69)	1 (1–2)	2.59	0.010
	≥20	2.47 (1.31)	2 (1–3)	1.63 (0.76)	1 (1–2)	2.93	0.003
**Psychological distress before COVID-19**	No	2.26 (1.09)	2 (1–3)	1.4 (0.63)	1 (1–2)	6.19	<0.001*
	Yes	3 (1.08)	3 (2–4)	1.95 (0.88)	2 (1–3)	4.13	<0.001*
**Previous experience with online learning **	No	2.51 (1.17)	2 (1.5–3)	1.53 (0.74)	1 (1–2)	6.90	<0.001*
	Yes	2.33 (0.92)	2 (2–3)	1.71 (0.81)	2 (1–2)	2.56	0.011
**Major life-changing due COVID-19**	No	2.48 (1.1)	2 (2–3)	1.53 (0.72)	1 (1–2)	6.71	<0.001*
	Yes	2.48 (1.27)	2 (1–3)	1.69 (0.89)	1 (1–2)	3.18	0.001
**Stress**							
**Gender **	Female	2.53 (1.2)	3 (1–3)	1.63 (0.83)	1 (1–2)	6.11	<0.001*
	Male	2.04 (1)	2 (1–3)	1.73 (0.92)	1 (1–2)	1.90	0.06
**Teaching experience (yrs.). **	< 20 years	2.49 (1.24)	3 (1–3)	1.73 (0.92)	1 (1–2)	4.63	<0.001*
	≥ 20 years	2.17 (1.02)	2 (1–3)	1.59 (0.78)	1 (1–2)	4.17	<0.001*
**Subject No.**	< 5 subjects	2.46 (1.2)	2 (1–3)	1.73 (0.9)	1 (1–2)	4.99	<0.001*
	≥ 5 subjects	2.14 (1.04)	2 (1–3)	1.55 (0.77)	1 (1–2)	3.86	<0.001*
**Weekly teaching hours (hrs.)**	5 to < 10	2.45 (1.14)	2 (2–3)	1.66 (0.94)	1 (1–2)	3.80	<0.001*
	10 to < 15	2.19 (1.16)	2 (1–3)	1.68 (0.83)	1 (1–2)	3.27	0.001
	15 to < 20	2.42 (1.26)	2 (1–3)	1.63 (0.83)	1 (1–2)	2.55	0.011
	≥20	2.47 (1.12)	2 (2–3)	1.68 (0.82)	1 (1–2)	2.80	0.005
**Psychological distress before COVID-19**	No	2.05 (1.08)	2 (1–3)	1.48 (0.73)	1 (1–2)	4.68	<0.001*
	Yes	3.1 (0.98)	3 (2–4)	2.12 (0.99)	2 (1–3)	4.08	<0.001*
**Previous experience with online learning**	No	2.37 (1.19)	2 (1–3)	1.63 (0.87)	1 (1–2)	5.75	<0.001*
	Yes	2.29 (1)	2 (1.5–3)	1.83 (0.82)	2 (1–2)	2.34	0.019
**Major life-changing due COVID-19**	No	2.33 (1.14)	2 (1–3)	1.65 (0.86)	1 (1–2)	5.58	<0.001*
	Yes	2.45 (1.21)	2 (1–3)	1.72 (0.88)	1 (1–2)	2.70	0.006
**Depression**							
**Gender**	Female	2.09 (1.15)	2 (1–3)	1.63 (1)	1 (1–2)	3.96	<0.001*
	Male	1.75 (0.91)	2 (1–2)	1.67 (0.86)	1 (1–2)	0.73	0.463
**Teaching experience (yrs.).**	< 20 years	1.99 (1.08)	2 (1–3)	1.54 (0.87)	1 (1–2)	3.87	<0.001*
	≥ 20 years	1.94 (1.09)	2 (1–3)	1.78 (1.04)	1 (1–3)	1.30	0.192
**Subject No.**	< 5 subjects	2.07 (1.19)	2 (1–3)	1.74 (1.03)	1 (1–3)	3.12	0.002
	≥ 5 subjects	1.76 (0.8)	2 (1–2)	1.47 (0.74)	1 (1–2)	2.33	0.020
**Weekly teaching hours (hrs.)**	5 to < 10	1.96 (1.13)	2 (1–3)	1.58 (0.91)	1 (1–2)	2.94	0.003
	10 to < 15	1.85 (0.95)	2 (1–2)	1.7 (1.01)	1 (1–2)	1.29	0.196
	15 to < 20	1.95 (1.27)	1 (1–3)	1.32 (0.67)	1 (1–1)	2.17	0.030
	≥20	2.32 (1.11)	2 (1–3)	2 (1.05)	2 (1–3)	1.22	0.221
**Psychological distress before COVID-19**	No	1.68 (0.91)	1 (1–2)	1.39 (0.71)	1 (1–2)	3.23	0.001
	Yes	2.67 (1.14)	3 (2–3)	2.26 (1.17)	2 (1–3)	2.15	0.032
**Previous experience with online learning **	No	1.96 (1.1)	2 (1–3)	1.62 (0.95)	1 (1–2)	3.64	<0.001*
	Yes	2 (0.98)	2 (1–3)	1.75 (0.94)	1 (1–2.5)	1.26	0.209
**Major life-changing due COVID-19**	No	1.89 (1.01)	2 (1–3)	1.63 (0.97)	1 (1–2)	0.30	0.003
	Yes	2.28 (1.31)	2 (1–3)	1.69 (0.89)	1 (1–2)	2.61	0.009

* significant at p<0.00104

Anxiety and stress levels significantly improved for the teachers regardless of their years of teaching experience, the number of subjects taught, or psychological distress before COVID-19. Teachers who had weekly teaching hours equal to or higher than 15 hrs. had a nonsignificant reduction in anxiety, while teachers who had weekly teaching hours equal to 10 hrs. or higher had a nonsignificant reduction in stress. Teachers without experience with online learning or who did not have major life-changing due to COVID-19 showed a significant reduction in anxiety and stress scores as time passed (Table 2). Also, a significant reduction in depression levels was observed in participants with experience of teaching for less than 20 years and without experience of remote teaching before the pandemic. In other groups, the reduction of reported depression levels was insignificant during the study (Table 2).

Moreover, the inter-frequencies analysis of stress and depression levels showed significant differences between teachers who reported having psychological distress before COVID-19 vs. teachers who declined this item, at the onset of the pandemic as well as post-pandemic. No other significant changes in frequencies were observed (Table 3).

**Table 3:** Inter-frequencies analysis between initial and current levels of anxiety, stress, and depression using Chi-square test of independence with Bonferroni correction.

Demographic parameter		Initial frequency				Current frequency			
		N (%)				N (%)			
		Normal /mild	Moderate/ more	Chi-square	p	Normal and mild	Moderate and more	Chi-square	p
Anxiety									
**Gender**	Female	44 (47.4)	49 (52.7)	2.396	0.122	80 (86.1)	13 (14)	0.141	0.707
** **									
	Male	31 (60.8)	20 (39.3)			45 (88.3)	6 (11.8)		
**Teaching experience (yrs.).**	< 20 years	38 (47)	43 (53.1)	1.983	0.159	69 (85.2)	12 (14.9)	0.424	0.515
** **	≥ 20 years	37 (58.8)	26 (41.3)			56 (88.9)	7 (11.2)		
**Subject No.**	< 5 subjects	49 (51.6)	46 (48.5)	0.028	0.866	82 (86.4)	13 (13.7)	0.058	0.809
	≥ 5 subjects	26 (53.1)	23 (47)			43 (87.8)	6 (12.3)		
**Weekly teaching hours (hrs.)**	5 to < 10	26 (49.1)	27 (51)	0.768	0.857	44 (83.1)	9 (17)	1.548	0.671
	10 to < 15	29 (54.8)	24 (45.3)			48 (90.6)	5 (9.5)		
	15 to < 20	9 (47.4)	10 (52.7)			17 (89.5)	2 (10.6)		
	≥20	11 (57.9)	8 (42.2)			16 (84.3)	3 (15.8)		
**Psychological distress before COVID-19**	No	62 (60.8)	40 (39.3)	10.609	0.001	94 (92.2)	8 (7.9)	8.744	0.003
	Yes	13 (31)	29 (69.1)			31 (73.9)	11 (26.2)		
**Previous experience with online learning**	No	61 (50.9)	59 (49.2)	0.451	0.502	104 (86.7)	16 (13.4)	0.012	0.912
	Yes	14 (58.4)	10 (41.7)			21 (87.5)	3 (12.5)		
**Major life-changing due COVID-19**	No	59 (51.3)	56 (48.7)	0.139	0.709	100 (87.0)	15 (13.0)	0.011	0.915
	Yes	16 (55.2)	13 (44.8)			25 (86.2)	4 (13.8)		
**Stress**									
**Gender**	Female	46 (49.5)	47 (50.6)	7.19	0.007	76 (81.8)	17 (18.3)	0.038	0.845
	Male	37 (72.6)	14 (27.5)			41 (80.4)	10 (19.7)		
**Teaching experience (yrs.).**	< 20 years	40 (49.4)	41 (50.7)	5.169	0.023	63 (77.8)	18 (22.3)	1.465	0.226
	≥ 20 years	43 (68.3)	20 (31.8)			54 (85.8)	9 (14.3)		
**Subject No.**	< 5 subjects	52 (54.8)	43 (45.3)	0.963	0.326	76 (80)	19 (20)	0.286	0.593
** **	≥ 5 subjects	31 (63.3)	18 (36.8)			41 (83.7)	8 (16.4)		
**Weekly teaching hours (hrs.)**	5 to < 10	30 (56.7)	23 (43.4)	0.878	0.831	44 (83.1)	9 (17)	0.242	0.971
	10 to < 15	33 (62.3)	20 (37.8)			43 (81.2)	10 (18.9)		
	15 to < 20	10 (52.7)	9 (47.4)			15 (79)	4 (21.1)		
	≥20	10 (52.7)	9 (47.4)			15 (79)	4 (21.1)		
**Psychological distress before COVID-19**	No	72 (70.6)	30 (29.5)	24.017	<0.001*	90 (88.3)	12 (11.8)	11.201	0.001
	Yes	11 (26.2)	31 (73.9)			27 (64.3)	15 (35.8)		
**Previous experience with online learning**	No	69 (57.5)	51 (42.5)	0.006	0.94	97 (80.9)	23 (19.2)	0.082	0.775
	Yes	14 (58.4)	10 (41.7)			20 (83.4)	4 (16.7)		
**Major life-changing due COVID-19**	No	67 (58.3)	48 (41.7)	0.09	0.764	94 (81.7)	21 (18.3)	0.09	0.765
	Yes	16 (55.2)	13 (44.8)			23 (79.3)	6 (20.7)		
**Depression**									
**Gender**	Female	61 (65.6)	32 (34.5)	4.544	0.033	75 (80.7)	18 (19.4)	0.100	0.707
	Male	42 (82.4)	9 (17.7)			40 (78.5)	11 (21.6)		
**Teaching experience (yrs.).**	< 20 years	59 (72.9)	22 (27.2)	0.156	0.692	68 (84)	13 (16.1)	1.925	0.165
	≥ 20 years	44 (69.9)	19 (30.2)			47 (74.7)	16 (25.4)		
**Subject No.**	< 5 subjects	65 (68.5)	30 (31.6)	1.323	0.250	71 (74.8)	24 (25.3)	4.558	0.033
	≥ 5 subjects	38 (77.6)	11 (22.5)			44 (89.8)	5 (10.3)		
**Weekly teaching hours (hrs.)**	5 to < 10	38 (71.7)	15 (28.4)	2.183	0.535	44 (83.1)	9 (17)	3.173	0.366
** **	10 to < 15	40 (75.5)	13 (24.6)			41 (77.4)	12 (22.7)		
	15 to < 20	14 (73.7)	5 (26.4)			17 (89.5)	2 (10.6)		
	≥20	11 (57.9)	8 (42.2)			13 (68.5)	6 (31.6)		
**Psychological distress before COVID-19**	No	84 (82.4)	18 (17.7)	20.123	<0.001*	91 (89.3)	11 (10.8)	19.028	<0.001*
	Yes	19 (45.3)	23 (54.8)			24 (57.2)	18 (42.9)		
**Previous experience with online learning**	No	86 (71.7)	34 (28.4)	0.007	0.934	97 (80.9)	23 (19.2)	0.423	0.515
	Yes	17 (70.9)	7 (29.2)			18 (75)	6 (25)		
**Major life-changing due COVID-19**	No	84 (73.0)	31 (27.0)	0.644	0.422	29 (80.0)	23 (20.0)	0.007	0.934
		19 (65.5)	10 (34.5)			23 (79.3)	6 (20.7)		

* significant at p<0.00119

### 3.3. Psychological distress predictors

Anxiety, stress, or depression were not significantly associated with years of experience, the number of subjects taught, and weekly teaching hours, but only with distress associated with academic work during COVID-19 (r_anxiety_ = 0.37, r_stress_ = 0.32, r_depression_ = 0.37, p<0.001). Moreover, they were positively correlated with the consideration or searching for psychological support because of the pandemic, experience of sleep disturbances because of pandemic rules, distress associated with constant canceling and reimplementation of the pandemic restrictions, and engagement in more unhealthy behaviors due to pandemic-associated distress (Table 4).

**Table 4:** Spearman rank order correlations between psychological distress predictors and anxiety, stress, and depression.

Question	Stress		Depression		Anxiety	
	Spearman	p sig.	Spearman	p sig.	Spearman	p sig.
	r		r		r	
Age	-0.169	0.043	0.003	0.972	-0.050	0.550
How many years have you worked as a teacher?	-0.156	0.061	0.032	0.707	-0.033	0.691
How many subjects do you teach?	-0.082	0.336	-0.113	0.185	-0.089	0.297
How many teaching hours per week do you have?	0.028	0.738	0.061	0.470	-0.033	0.696
Distress associated with academic work during the COVID-19 pandemic was higher than before.	0.367	<0.001*	0.322	<0.001*	0.370	<0.001*
I considered and/or actively sought out psychological support because of distress (anxiety, fear, fatigue, depression,) due to pandemic	0.458	<0.001*	0.436	<0.001*	0.456	<0.001*
I experienced sleep disturbances since the introduction of the pandemic rules	0.434	<0.001*	0.364	<0.001*	0.453	<0.001*
Distress during the COVID-19 pandemic was lowered after cancelling some restrictions	0.022	0.796	0.073	0.387	0.027	0.749
Constant cancelling and reimplementation of COVID restrictions has contributed to an increase in stress	0.276	0.001*	0.253	0.002	0.287	<0.001*
Distress during the COVID-19 pandemic was lowered with the passing time	0.034	0.687	0.018	0.826	0.048	0.565
Distress during the COVID-19 pandemic was lowered after implementing well-organized remote teaching	-0.023	0.783	-0.058	0.491	0.048	0.565
Distress during the COVID-19 pandemic was lowered after receiving COVID vaccination (if vaccinated)	-0.009	0.912	0.053	0.526	0.121	0.147
Distress during the COVID-19 pandemic was lowered after COVID infection (if infected)	-0.048	0.568	-0.077	0.358	0.009	0.914
I engaged in more unhealthy behaviors during the COVID-19 pandemic due to experienced distress	0.431	<0.001*	0.366	<0.001*	0.319	<0.001*
It was easy for me to adapt to virtual learning	0.036	0.667	-0.095	0.255	-0.022	0.790

* For multiple comparisons, significant for absolute correlation coefficient higher than 0.269.

### 3.4. Virtual learning concerns

For the virtual learning concerns, almost 79% of teachers reported that students might engage less; this opinion was correlated with higher weekly teaching hours (r = 0.19, p<0.05). Cheating was the least reported concern and was associated with more subjects (r = 0.20, p<0.05). Finally, we found a positive correlation between major life-change due to COVID-19 and the occurrence of technical difficulties (r = 0.24, p<0.05). The major online learning concern frequencies and correlation list is shown in Table 5.

**Table 5:** Spearman rank order correlations between major online learning concerns and demographic parameters.

Demographic parameter	Major online learning concerns				
	Unable to follow-up with the students (35.4%)	Students will cheat (29.9%)	Students will not pay attention (64.6%)	Technical difficulties during classes (36.1%)	Students participate/engage less (79.2%)
**Gender** ^**Φ**^	-0.032	-0.039	-0.028	0.018	0.022
**Teaching experience (yrs.).**	0.047	-0.017	0.026	-0.070	-0.025
**Subject No.**	0.051	0.202*	0.117	0.106	0.084
**Weekly teaching hours (hrs.)**	0.030	0.063	0.082	0.028	0.192*
**Psychological distress before COVID-19**	-0.028	0.149	-0.068	0.027	-0.085
**Remote teaching before COVID-19** ^**Φ**^	-0.058	-0.088	-0.019	0.052	-0.138
**Major life-changing due COVID-19** ^**Φ**^	0.063	0.013	0.118	0.235*	0.130

* Significant at p<0.05

Φ Calculated using phi coefficient

### 3.5. Multivariate models

In the multivariate analysis (Table 6), improvement of anxiety has been associated with being female (OR = 0.45; 95% CI = -1.58-(-0.03); p = 0.04), lowered distress with passing time (OR = 2.7; 95%CI = 0.49–1.50; p<0.001), and lowered distress due to COVID-19 infection (OR = 1.25; 95%CI = 0.001–0.44; p = 0.049).

**Table 6:** Multivariate models for improved levels of anxiety, stress, and depression.

	Estimate	Standard Error	Wald Stat.	95.0% CL		p sig.	OddsRatio
				Lower	Upper		
Anxiety							
**Gender**	-0.808	0.395	4.174	-1.583	-0.033	0.041	0.446
**Distress during the COVID-19 pandemic was lowered with the passing time**	0.996	0.256	15.129	0.494	1.497	<0.001	2.706
**Distress during the COVID-19 pandemic was significantly lowered after COVID infection (if infected)**	0.219	0.111	3.877	0.001	0.437	0.049	1.245
**Stress**							
**Gender**	-1.024	0.409	6.270	-1.826	-0.223	0.012	0.359
**Distress during the COVID-19 pandemic was lowered with the passing time**	0.851	0.274	9.630	0.313	1.388	0.002	2.341
**I experienced sleep disturbances since the introduction of the pandemic rules**	0.358	0.165	4.698	0.034	0.682	0.030	1.431
**Depression**							
**Gender**	-0.989	0.441	5.025	-1.854	-0.124	0.025	0.372
**Years of experience**	-0.919	0.401	5.245	-1.705	-0.132	0.022	0.399
**Distress during the COVID-19 pandemic was lowered with the passing time**	0.828	0.301	7.561	0.238	1.419	0.006	2.290
**I experienced sleep disturbances since the introduction of the pandemic rules**	0.327	0.162	4.046	0.008	0.645	0.044	1.386

Likewise, better stress scores were associated with being female (OR = 0.36; 95%CI = -1.83- (-0.22); p = 0.01), lowered distress with passing time (OR = 2.34; 95%CI = 0.31–1.39; p = 0.002) and experiencing sleep disturbances since the introduction of the pandemic rules (OR = 1.43; 95%CI = 0.03–0.68; p = 0.03).

Finally, improved depression level could be predicted by gender (OR = 0.37; 95%CI = -1.85-(-0.12); p = 0.03), years of experience—only those below 20 years of experience were most likely to report improvement in depression—(OR = 0.4; 95% CI = -1.71-(-0.13); p = 0.02); lowered distress during the pandemic with passing time (OR = 2.29; 95%CI = 0.24-(1.42); p = 0.006) and experiencing sleep disturbances (OR = 1.39; 95% CI = 0.008–0.64; p = 0.04).

## 4. Discussion

This study set out to examine effects of the COVID-19 pandemic on psychological distress experienced by Polish academic medical school teachers. Our findings show that anxiety levels have significantly decreased across all varying factors. Females are more prone to stress (more exactly: to react more intense to a stressor) than men. According to the multivariate model, females who reported lowered distress after a COVID-19 infection, sleep disturbances, and decreased distress over time are predicted to have lower anxiety, stress, and depression levels.

### 4.1. Age

Age was not significantly associated with anxiety, stress, or depression in our study. However, age (and therefore experience of the teacher) descriptively correlated negatively with stress (i.e. older and more experienced teachers feel less stress), which is quite plausible; relatively young teachers may have concerns about their future career, scientific mobility, clinical rotations, and specialization. There is basically no association between experience (as measured by biological age and/or teaching experience) and depression or anxiety levels. However, the age of the teachers in this sample was 44.85 ± 10.78 years which may reflect academic career stability. In contrast, a study conducted in Spain with a sample size of 1,633 teachers revealed that anxiety and stress were more prevalent among women and older participants (>47 years) [9]. These findings were also confirmed by the fact that studies conducted pre-pandemic showed that anxiety and stress were higher among women due to the role of a caregiver (both personally and professionally), which increased the symptomatology [9]. In contrast to other studies, it was reported that older teachers (>47 years) experienced higher levels of anxiety and stress due to difficulties that may have arisen as a consequence of adapting to new technologies when compared to younger teachers [9].

### 4.2. Gender

We found that females experienced mild to moderate levels of anxiety, stress, and depression at the beginning of the pandemic, which was significantly reduced after COVID-19-Pandemic restrictions were lifted (2 years later). Male participants displayed a constant level of reported depression (floor effect) from the start of the pandemic to post-pandemic state, which could be explained by generally low reported levels of depression in this group. Even though their anxiety and stress levels were lower in the post-pandemic state, the differences were insignificant, which could be clinically relevant but not be detected due to the low sample size.

It is worth mentioning that females reported significantly (p = 0.013) higher anxiety levels than men at the beginning of the COVID-19 pandemic (2.66 vs. 2.16), while they reported numerically lower anxiety levels in the post-pandemic stages (1.55 vs. 1.59). Additionally, men reported significantly (p = 0.019) lower stress levels than females at the beginning of the COVID-19 pandemic (2.04 vs. 2.53), while they reported numerically higher stress levels in the post-pandemic stages (1.73 vs. 1.63). No significant differences were observed between males and females in depression at the beginning of the COVID-19 pandemic or in the post-pandemic stages.

These results suggest that females seem to be affected more by the stressor than men but recover to similar levels as men when the stressor is removed. They also may indicate the ability of women to handle psychological distress actively and progressively compared to men, such as seeking help or employing more efficient coping mechanisms. Similar results were obtained after evaluating 105 academic teachers in the Philippines; women expressed higher stress levels while teaching during the pandemic (p = 0.037) [10].

### 4.3. Experience

According to Table 2, teachers with less than 20 years of experience reported numerically higher anxiety (2.6 vs. 2.32), higher stress (2.49 vs. 2.17), and minimally higher depression (1.99 vs. 1.94) during the initial phase of COVID-19. Even though these differences were not statistically significant, they may indicate the critical role of experience in facing sudden situations. It is also worth adding that teachers with less than 20 years of experience were more prone to depression than those with 20 years or more of experience. In Chile, physical function, bodily pain, vitality, and mental health were assessed among 336 teachers, in which women and individuals <44 years of age showed the lowest score (p< 0.01). The authors claimed that older teachers can problem solve independently and are more competent in their routine work. Hence, mental health deterioration was observed among teachers below 44 years old [11].

### 4.4. Teaching hours

In our study, academic teachers with less than 15 weekly teaching hours reported a significant reduction in anxiety when comparing the time of pandemic onset and the moment of the study (Table 2). Even though teaching hours were not correlated with anxiety, stress, or depression, the workload during COVID-19 showed a relation to such emotions, as reported by 48% of the teachers, see Table 4. As reported by Baker et al. [12], after transitioning to remote learning, teachers (N = 444) experienced an overload of work requiring them to be available at any time of the day with no ‘real’ working hours [12]. However, few studies investigated correlations between teaching hours and psychological distress. Academic teachers in Jordan (N = 299) needed 4.4 hours per day to prepare for remote classes after the onset of the pandemic [13]. Another study (N = 382) showed that teachers had to spend more time preparing assignments and examinations and be ready to answer students’ questions, which led to a disturbance in leisure time [8]. German teachers (N = 394) spent over 4 hours per day on remote teaching, which was associated with significantly higher stress in comparison with teachers working less (r = 0.41, p<0.001) [14]. In Chile, distress among teachers (N = 336) was associated with working more than before the pandemic [11] and for more than one additional unpaid hour per day [15]. In Spain, the emotional problems of teachers (N = 345) were predicted positively by the number of teaching hours [16].

### 4.5. Online learning

Regardless of digital expertise level, teachers were required to use online learning tools, which may have been overwhelming [17]. We did not notice significant differences in anxiety, stress, or depression levels between the teachers with versus without previous experience with online learning. However, those without previous experience with online learning showed significant improvement in anxiety, stress, and depression levels in the post-pandemic stage (Table 2). It is worth mentioning that even though only 16.7% reported previous experience with online teaching, academic medical teachers are using the computer and various technologies daily. Therefore, 62% have reported easy adaptation to online learning. Still, several concerns have been raised. Almost 80% of the teachers reported “students participate/engage less” as the primary concern, and it was associated with weekly teaching hours (r = 0.19, p<0.05). Although cheating was the least reported concern (29.9%), it was associated with the number of subjects taught (r = 0.2, p<0.05) (Table 5). Similar results were reported by Sokolová et al. after surveying 600 secondary school and university teachers across Europe [18]. They reported that increased workload with online learning was the most challenging barrier reported by 64.7% of the teachers because the changes from face-to-face to online learning were sudden, and teachers were unprepared for this condition. Hence, they required time to search for suitable software and sources [18]. Moreover, poor internet connections and lack of engagement and motivation from students were reported by 50.76 and 36.82% of the teachers [18].

Other studies investigating teachers’ online learning experiences have shed light on multiple factors that have contributed to psychological distress and those that have alleviated the sudden transition to E-teaching challenges. A cross-sectional study involving teachers all over India showed that switching to online classes has led to consequences involving physical and mental discomforts such as headache, eye strain, and less satisfaction due to lack of personal contact. Furthermore, 44% of school teachers suffered mental or physical discomfort, and the online learning method was uncomfortable for 87.5% of the respondents [4]. Classes were said to be more monotonous and less motivating to teach, and teaching struggling students was even more challenging, all of which added more psychological distress to the teachers [4].

A mixed-methods study involving 107 teachers throughout the United States revealed several challenges teachers face, such as learning new technology without training, lack of face-to-face interactions with students, worse work-life balance, and declined student engagement during class. Amidst these challenges, 51% of teachers perceived online teaching as challenging yet rewarding, with 44% finding it enjoyable and 60% finding it stressful. Almost 81% of the teachers preferred in-person teaching over virtual [19]. Another study (N = 394) showed that previous training and experience with online teaching decreased distress and perceived stress among teachers in Ecuador, suggesting a possible relationship between psychological distress, preparedness, and online skill level [20]. It may thus be extrapolated that a significant source of psychological distress has been attributed to teachers’ lack of preparedness in the face of escalated use of online teaching and the lack of skills needed for a comfortable transition. This may also explain why most studies have found teachers to prefer traditional face-to-face teaching, although the absence of the inherent human need for interpersonal connection during online teaching may play a more prominent role [21]. In an Italian study (N = 97), approximately 50% of academics experienced difficulties getting familiar with e-learning platforms, and 43.5% reported network problems or hardware issues [22]. Since the start of online classes, 36% of teachers have noticed a decrease in student attendance and engagement during courses, and 21% felt a loss of personal contact with their students in a study conducted within 14 universities in the UK [23]. Academics reported that they find it increasingly challenging to interact with the students during class discussions, even more so without a webcam [24]. A study in Jordan showed that teachers were exhausted by the amount of work needed to be invested in planning and conducting online classes and preparing examinations remotely. Eighty-three percent of the teachers also worried about the increased possibility of students cheating in online examinations without using any proctored portals [8]. Overall, online teaching is perceived to be efficient, yet it is not the preferred method [19].

### 4.6. Major life changes

Even though 20% of the participants reported having major changes in their life due to COVID-19, such as the death of a close one, moving to a different country, or losing a job, we did not observe any relationship between life changes and depression, but it influenced anxiety and stress levels (Table 2) without impacting the teaching experience (Table 5). Having a stable job and/or being resistant to stressful situations due to its nature could be factors that explain why no relationship was found. Other studies have reported different results; a qualitative study conducted at the beginning of the pandemic in France observed psychological distress experienced by family members of 12 ICU Covid-19 positive patients [25]. Due to the unfortunate circumstances, family members of the deceased were unable to see or hold their loved ones for the last time due to health and safety guidelines that required health authorities to forbid access to body bags [25]. In some situations, final rituals were unable to be performed [25]. Many families (16 interviews) described the experience as dehumanizing and, consequently, hindered their grieving process [25]. In a longitudinal study involving 9,024 Brazilians, those who experienced a loss of a loved one due to COVID-19 had amplified psychological distress [26].

Moreover, an Australian prospective longitudinal cohort study involving 2,603 respondents used the Kessler Psychological Distress Scale to measure psychological distress among groups of people with various pandemic-related job outcomes [27]. The groups that experienced work loss suffered more mentally than physically, with higher odds of high psychological distress (adjusted OR = 2.22–3.66) than those whose jobs remained. For instance, 30% of the respondents who lost their jobs showed high psychological distress (K-6 score >18) compared to only 1.9% of the unaffected employees. Interestingly, this effect was moderated by other factors such as social interaction and financial resources—less social interaction and higher financial hardship exacerbated the psychological distress caused by job loss (adjusted OR = 5.43–8.36) [27].

### 4.7. The need for psychological support

A policy brief by the Organization for Economic Co-operation and Development (OECD) described that in 10 countries from several continents, the anxiety and depression levels observed in citizens in the year 2020 were more than twice as high as levels measured in previous years, being exceptionally high throughout the COVID outbreak in March 2020. During the lockdown, new forms of psychological support were demanded, such as online psychological counseling, video conferences, or telephone hotlines [28].

In our study, we noticed that teachers with psychological distress before COVID-19 reported significantly (p<0.001) higher levels of anxiety (3 vs. 2.26), stress (3.1 vs. 2.05), and depression (2.67 vs. 1.68) than teachers without psychological distress (Table 2). Even though levels of anxiety and stress have improved in teachers with psychological distress at the time of the study, their levels of anxiety, stress, and depression are still significantly (p<0.001) higher than teachers without psychological distress [(1.95 vs. 1.4), (2.12 vs. 1.48), and (2.26 vs. 1.39), respectively].

A survey conducted in Lithuania about psychological support offered to the public (N = 240) revealed that 89.2% of respondents believed the need for such support during the pandemic has increased. They also stated that the available psychological services are not sufficient and poorly organized [29]. In a study performed in 2018, before the onset of the pandemic (N = 603), 54% of Chinese university teachers reached a positive rate (over 21 points) on the 10-item Kessler Psychological Distress Scale (K10), indicating experience of distress [5]. In our study, only 29,2% of responders reported having psychological distress before the COVID-19 pandemic, but it may be underrated due to retrospective assessment. Moreover, reporting higher distress after the pandemic onset and seeking psychological support were correlated positively with the reported level of anxiety, stress, and depression (Table 4).

According to Steinhardt et al., chronic stress may lead to depression and symptoms of burnout [30]. Furthermore, teachers’ well-being and care for mental health positively impact their students’ academic self-perception, well-being, and potential distress [31, 32]. It is essential to support teachers with skills to prevent mental health deterioration [33], and many teachers supporting models have been proposed [32, 34, 35]. Garcia-Alvarez et al. indicated the importance of active interventions based on positive psychology in private and professional life and the promotion of well-being approaches [36]. However, there is a lack of high-quality studies concerning interventions to reduce teachers’ distress [37]. This highlights the need for robust psychological support programs for proper intervention at universities.

## 5. Strengths and limitations

We performed a cross-sectional study and showed a significant distress change associated with the COVID-19 pandemic. To our knowledge, it is the first study assessing the psychological distress among academic teachers in Poland after two years of the COVID-19 pandemic—at the time of withdrawal of almost all restrictions. Moreover, having all participants from a medical university may increase the quality of the response, especially those considered self-reporting. However, we admit that our study has limitations. Our responses were collected from academic teachers from only one university. The measures were made retrospectively, and we used only single item measures of psychological concepts. Also, the responses we received do not represent academic teachers overall. The participants are medical teachers, which means there could be a higher awareness of mental illness/distress, so it could be that our respondents have better coping skills than average academic teachers.

## 6. Conclusion

Our results demonstrated the influence of gender and COVID-19 duration on anxiety, stress, and depression. Females recorded higher scores on the three scales, while overall, the scores reduced with time. Reduced distress after COVID-19 infection was associated with improved anxiety status. Surprisingly, anxiety, stress, or depression were not significantly associated with years of experience, the number of taught subjects, and weekly teaching hours but only with the academic work during COVID-19, which may indicate better coping skills among academic medical teachers. Most importantly, levels of anxiety, stress, or depression were not significantly associated with any virtual learning concerns. Further research is required to address the coping skills of academic medical teachers and to identify other factors associated with anxiety, stress, and depression, such as socioeconomic status, administrative roles, annual publication records, grants availability, medical specialty, and clinical practicing hours. We recommend that medical universities create, maintain and improve psychological support programs post-pandemic.

## Supporting information

S1 File Survey file. (DOCX)

S1 Data (XLSX)

S1 Graphical abstract (TIF)

## References

[1] Kalish H, Klumpp-Thomas C, Hunsberger S, Baus HA, Fay MP, Siripong N, et al. Undiagnosed SARS-CoV-2 seropositivity during the first 6 months of the COVID-19 pandemic in the United States. Sci Transl Med. 2021;13: eabh3826. doi: 10.1126/scitranslmed.abh3826 34158410 PMC8432952

[2] Haleem A, Javaid M, Vaishya R. Effects of COVID-19 pandemic in daily life. Current Medicine Research and Practice. 2020;10: 78–79. doi: 10.1016/j.cmrp.2020.03.011 32292804 PMC7147210

[3] Ozamiz-Etxebarria N, Idoiaga Mondragon N, Bueno-Notivol J, Pérez-Moreno M, Santabárbara J. Prevalence of Anxiety, Depression, and Stress among Teachers during the COVID-19 Pandemic: A Rapid Systematic Review with Meta-Analysis. Brain Sciences. 2021;11: 1172. doi: 10.3390/brainsci11091172 34573192 PMC8468121

[4] Selvaraj A, Radhin V, KA N, Benson N, Mathew AJ. Effect of pandemic based online education on teaching and learning system. International Journal of Educational Development. 2021;85: 102444. doi: 10.1016/j.ijedudev.2021.102444 34518732 PMC8426326

[5] Li W, Kou C. Prevalence and correlates of psychological stress among teachers at a national key comprehensive university in China. International Journal of Occupational and Environmental Health. 2018;24: 7–16. doi: 10.1080/10773525.2018.1500803 30047833 PMC6225434

[6] Besser A, Lotem S, Zeigler-Hill V. Psychological Stress and Vocal Symptoms Among University Professors in Israel: Implications of the Shift to Online Synchronous Teaching During the COVID-19 Pandemic. Journal of Voice. 2020; S0892199720301909. doi: 10.1016/j.jvoice.2020.05.028 32600872 PMC7274605

[7] Moser KM, Wei T, Brenner D. Remote teaching during COVID-19: Implications from a national survey of language educators. System. 2021;97: 102431. doi: 10.1016/j.system.2020.102431

[8] Akour A, Al-Tammemi AB, Barakat M, Kanj R, Fakhouri HN, Malkawi A, et al. The Impact of the COVID-19 Pandemic and Emergency Distance Teaching on the Psychological Status of University Teachers: A Cross-Sectional Study in Jordan. The American Journal of Tropical Medicine and Hygiene. 2020;103: 2391–2399. doi: 10.4269/ajtmh.20-0877 33124547 PMC7695050

[9] Ozamiz-Etxebarria N, Berasategi Santxo N, Idoiaga Mondragon N, Dosil Santamaría M. The Psychological State of Teachers During the COVID-19 Crisis: The Challenge of Returning to Face-to-Face Teaching. Front Psychol. 2021;11: 620718. doi: 10.3389/fpsyg.2020.620718 33510694 PMC7835279

[10] Oducado RM, Rabacal J, Moralista R, Tamdang K. Perceived Stress Due COVID-19 Pandemic Among Employed Professional Teachers. ijeri. 2020; 305–316. doi: 10.46661/ijeri.5284

[11] Lizana PA, Vega-Fernadez G. Teacher Teleworking during the COVID-19 Pandemic: Association between Work Hours, Work–Family Balance and Quality of Life. IJERPH. 2021;18: 7566. doi: 10.3390/ijerph18147566 34300015 PMC8304294

[12] Baker CN, Peele H, Daniels M, Saybe M, Whalen K, Overstreet S, et al. The Experience of COVID-19 and Its Impact on Teachers’ Mental Health, Coping, and Teaching. School Psychology Review. 2021;50: 491–504. doi: 10.1080/2372966X.2020.1855473

[13] Almhdawi KA, Obeidat D, Kanaan SF, Hajela N, Bsoul M, Arabiat A, et al. University professors’ mental and physical well-being during the COVID-19 pandemic and distance teaching. WOR. 2021;69: 1153–1161. doi: 10.3233/WOR-205276 34420997

[14] Federkeil L, Heinschke F, Jungmann T, Klapproth F. Teachers experiences of stress and their coping strategies during COVID—19 induced distance teaching. JPR. 2020;4: 444–452. doi: 10.33902/JPR.2020062805

[15] Palma-Vasquez C, Carrasco D, Hernando-Rodriguez JC. Mental Health of Teachers Who Have Teleworked Due to COVID-19. EJIHPE. 2021;11: 515–528. doi: 10.3390/ejihpe11020037 34708828 PMC8314372

[16] Aperribai L, Cortabarria L, Aguirre T, Verche E, Borges Á. Teacher’s Physical Activity and Mental Health During Lockdown Due to the COVID-2019 Pandemic. Front Psychol. 2020;11: 577886. doi: 10.3389/fpsyg.2020.577886 33262727 PMC7685995

[17] Pokhrel S, Chhetri R. A Literature Review on Impact of COVID-19 Pandemic on Teaching and Learning. Higher Education for the Future. 2021;8: 133–141. doi: 10.1177/2347631120983481

[18] Sokolová L, Papageorgi I, Dutke S, Stuchlíková I, Williamson M, Bakker H. Distance Teaching of Psychology in Europe: Challenges, Lessons Learned, and Practice Examples During the First Wave of COVID-19 Pandemic. Psychology Learning & Teaching. 2022;21: 73–88. doi: 10.1177/14757257211048423

[19] An Y, Kaplan-Rakowski R, Yang J, Conan J, Kinard W, Daughrity L. Examining K-12 teachers’ feelings, experiences, and perspectives regarding online teaching during the early stage of the COVID-19 pandemic. Educational Technology Research and Development. 2021;69: 2589–2613. doi: 10.1007/s11423-021-10008-5 34220171 PMC8237773

[20] Hidalgo-Andrade P, Hermosa-Bosano C, Paz C. Teachers’ Mental Health and Self-Reported Coping Strategies During the COVID-19 Pandemic in Ecuador: A Mixed-Methods Study. Psychology Research and Behavior Management. 2021;Volume 14: 933–944. doi: 10.2147/PRBM.S314844 34239334 PMC8259946

[21] Fauzi I, Sastra Khusuma IH. Teachers’ Elementary School in Online Learning of COVID-19 Pandemic Conditions. Jurnal Iqra’: Kajian Ilmu Pendidikan. 2020;5: 58–70. doi: 10.25217/ji.v5i1.914

[22] Casacchia M, Cifone MG, Giusti L, Fabiani L, Gatto R, Lancia L, et al. Distance education during COVID 19: an Italian survey on the university teachers’ perspectives and their emotional conditions. BMC Med Educ. 2021;21: 335. doi: 10.1186/s12909-021-02780-y 34107926 PMC8187887

[23] Longhurst GJ, Stone DM, Dulohery K, Scully D, Campbell T, Smith CF. Strength, Weakness, Opportunity, Threat (SWOT) Analysis of the Adaptations to Anatomical Education in the United Kingdom and Republic of Ireland in Response to the Covid‐19 Pandemic. Anat Sci Educ. 2020;13: 301–311. doi: 10.1002/ase.1967 32306550 PMC7264742

[24] Tsai C-H, Romera Rodriguez G, Li N, Robert J, Serpi A, Carroll JM. Experiencing the Transition to Remote Teaching and Learning during the COVID-19 Pandemic. IxD&A. 2020; 70–87. doi: 10.55612/s-5002-046-004

[25] Kentish-Barnes N, Cohen-Solal Z, Morin L, Souppart V, Pochard F, Azoulay E. Lived Experiences of Family Members of Patients With Severe COVID-19 Who Died in Intensive Care Units in France. JAMA Netw Open. 2021;4: e2113355. doi: 10.1001/jamanetworkopen.2021.13355 34152418 PMC8218069

[26] Joaquim RM, Pinto ALCB, Guatimosim RF, de Paula JJ, Souza Costa, Diaz AP, et al. Bereavement and psychological distress during COVID-19 pandemics: The impact of death experience on mental health. Current Research in Behavioral Sciences. 2021;2: 100019. doi: 10.1016/j.crbeha.2021.100019

[27] Griffiths D, Sheehan L, Van Vreden C, Petrie D, Grant G, Whiteford P, et al. The Impact of Work Loss on Mental and Physical Health During the COVID-19 Pandemic: Baseline Findings from a Prospective Cohort Study. Journal of Occupational Rehabilitation. 2021;31: 455–462. doi: 10.1007/s10926-021-09958-7 33656699 PMC7926060

[28] OECD. Tackling the mental health impact of the COVID-19 crisis: An integrated, whole-of-society response. 2021. Available: https://www.oecd-ilibrary.org/content/paper/0ccafa0b-en

[29] Davulis T, Gasparėnienė L, Raistenskis E. Assessment of the situation concerning psychological support to the public and business in the extreme conditions: case of Covid-19. Entrepreneurship and sustainability issues. 2021;8: 308–321. doi: 10.9770/jesi.2021.8.3(19)

[30] Steinhardt MA, Smith Jaggars SE, Faulk KE, Gloria CT. Chronic Work Stress and Depressive Symptoms: Assessing the Mediating Role of Teacher Burnout: Stress, Burnout and Depressive Symptoms. Stress and Health. 2011;27: 420–429. doi: 10.1002/smi.1394

[31] Carroll A, York A, Fynes-Clinton S, Sanders-O’Connor E, Flynn L, Bower JM, et al. The Downstream Effects of Teacher Well-Being Programs: Improvements in Teachers’ Stress, Cognition and Well-Being Benefit Their Students. Front Psychol. 2021;12: 689628. doi: 10.3389/fpsyg.2021.689628 34276519 PMC8281271

[32] Harding S, Morris R, Gunnell D, Ford T, Hollingworth W, Tilling K, et al. Is teachers’ mental health and wellbeing associated with students’ mental health and wellbeing? Journal of Affective Disorders. 2019;242: 180–187. doi: 10.1016/j.jad.2018.08.080 30189355

[33] Greer JG, Greer BB. Stopping Burnout Before it Starts: Prevention Measures at the Preservice Level. Teacher Education and Special Education. 1992;15: 168–174. doi: 10.1177/088840649201500303

[34] Skinner E, Beers J. Mindfulness and Teachers’ Coping in the Classroom: A Developmental Model of Teacher Stress, Coping, and Everyday Resilience. In: Schonert-Reichl KA, Roeser RW, editors. Handbook of Mindfulness in Education. New York, NY: Springer New York; 2016. pp. 99–118. doi: 10.1007/978-1-4939-3506-2_7

[35] Hepburn S-J, Carroll A, McCuaig L. Exploring a Complementary Stress Management and Wellbeing Intervention Model for Teachers: Participant Experience. IJERPH. 2021;18: 9009. doi: 10.3390/ijerph18179009 34501595 PMC8430586

[36] García-Álvarez D, Soler MJ, Achard-Braga L. Psychological Well-Being in Teachers During and Post-Covid-19: Positive Psychology Interventions. Frontiers in Psychology. 2021;12. Available: https://www.frontiersin.org/articles/10.3389/fpsyg.2021.769363 10.3389/fpsyg.2021.769363 PMC8716601 34975659

[37] Naghieh A, Montgomery P, Bonell CP, Thompson M, Aber JL. Organisational interventions for improving wellbeing and reducing work-related stress in teachers. Cochrane Work Group, editor. Cochrane Database of Systematic Reviews. 2015 [cited 19 Apr 2022]. doi: 10.1002/14651858.CD010306.pub2 25851427 PMC10993096

