# Supplementary material for: Changes in psychological distress among Polish medical university teachers during the COVID-19 pandemic
Source: PLoS One. 2022 Dec 1;17(12):e0278311. doi: 10.1371/journal.pone.0278311 (PMC9714882; doi:10.1371/journal.pone.0278311)
Supplement: S1 File — (DOCX) [file pone.0278311.s001.docx]

| **Demographic data** | |
| --- | --- |
| Age |  |
| Gender |  |
| How many years have you worked as a teacher? |  |
| How many subjects do you teach? |  |
| How many teaching hours per week do you have? | 5 to <10 hours a week  10 to <15 hours a week  15 to <20 hours a week  ≥20 hours a week |
| Have you struggled with psychological distress (i.e. stress, depression, anxiety, PTSD) prior to the COVID-19 pandemic? | Yes  No |
| Before COVID-19, have you ever taught a class via remote techniques? | Yes  No |
| Has the COVID-19 pandemic led to any major life-changing events (death of a close one, moving to a different country, losing a job, etc). | Yes  No |
| **Anxiety, stress and depression scale** | |
| On a scale of 1 to 5, please estimate your anxiety level (1 being Normal and 5 being extremely severe)  Anxiety: If you have an anxiety disorder, you may experience:  Fear, panic or anxiety in situations where most people would not feel anxious or threatened  A constant nagging worry or anxiousness  Sudden panic or anxiety attacks without any clear trigger | When COVID-19 was first discovered  Normal  Mild  Moderate  Severe  Extremely severe  Currently  Normal  Mild  Moderate  Severe  Extremely severe |
| On a scale of 1 to 5, please estimate your stress level (1 being Normal and 5 being extremely severe)  Stress affects a lot of people, and can influence your health. Symptoms include:  Headaches  High blood pressure  Chest pain  Heart palpitations  Skin rashes  Loss of sleep  Stress typically goes away when the stressors disappear | When COVID-19 was first discovered  Normal  Mild  Moderate  Severe  Extremely severe  Currently  Normal  Mild  Moderate  Severe  Extremely severe |
| On a scale of 1 to 5, please estimate your Depression level (1 being Normal and 5 being extremely severe)  Depression: When you’re depressed, it affects just about everything in your life — how you think, feel, behave and function. You may experience one or more of these symptoms:  Discouragement  Sadness  Hopelessness  Anger  Lack of motivation or interest in life in general  Low energy level  Insomnia  Feeling overwhelmed by daily tasks and personal interactions | When COVID-19 was first discovered  Normal  Mild  Moderate  Severe  Extremely severe  Currently  Normal  Mild  Moderate  Severe  Extremely severe |
| **Predictors of psychological distress** | |
| Distress associated with academic work during the COVID-19 pandemic was higher than before its onset. | Strongly disagree  Disagree  Neutral  Agree  Strongly agree |
| I considered and/or actively sought out psychological support because of distress (anxiety, fear, fatigue, depression, etc) during the COVID-19 pandemic | Strongly disagree  Disagree  Neutral  Agree  Strongly agree |
| I experienced sleep disturbances since the introduction of the pandemic rules | Strongly disagree  Disagree  Neutral  Agree  Strongly agree |
| Distress during the COVID-19 pandemic was lowered after cancelling some restrictions | Strongly disagree  Disagree  Neutral  Agree  Strongly agree |
| Constant cancelling and reimplementation of COVID restrictions has contributed to an increase in stress | Strongly disagree  Disagree  Neutral  Agree  Strongly agree |
| Distress during the COVID-19 pandemic was lowered with the passing time | Strongly disagree  Disagree  Neutral  Agree  Strongly agree |
| Distress during the COVID-19 pandemic was significantly lowered after implementing well-organized remote teaching | Strongly disagree  Disagree  Neutral  Agree  Strongly agree |
| Distress during the COVID-19 pandemic was significantly lowered after receiving COVID vaccination (if vaccinated) | Strongly disagree  Disagree  Neutral  Agree  Strongly agree  Not vaccinated |
| Distress during the COVID-19 pandemic was significantly lowered after COVID infection (if infected) | Strongly disagree  Disagree  Neutral  Agree  Strongly agree  Not infected |
| I engaged in more unhealthy behaviours during the COVID-19 pandemic due to experienced distress | Strongly disagree  Disagree  Neutral  Agree  Strongly agree |
| It was easy for me to adapt to virtual learning | Strongly disagree  Disagree  Neutral  Agree  Strongly agree |
| What are your major concerns about the remote learning? (Select all that applies) | Unable to follow-up with the students  Students will cheat  Students will not pay attention  Technical difficulties during classes  Students participate/engage less |
