# Supplementary figures and images for: Changes in psychological distress among Polish medical university teachers during the COVID-19 pandemic
Source: PLoS One. 2022 Dec 1;17(12):e0278311. doi: 10.1371/journal.pone.0278311 (PMC9714882; doi:10.1371/journal.pone.0278311)

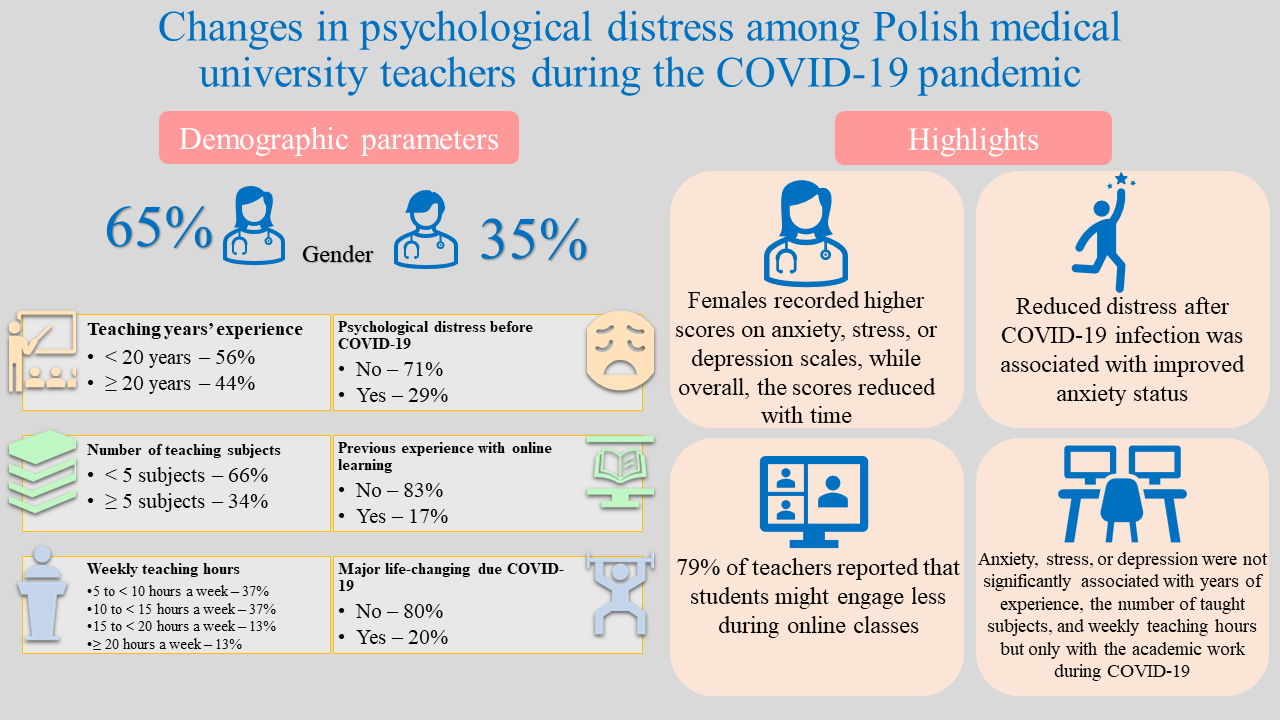

Supplement: S1 Graphical abstract — (TIF) [file pone.0278311.s003.tif]
